# Functional Consequences of the Postnatal Switch From Neonatal to Mutant Adult Glycine Receptor α1 Subunits in the *Shaky* Mouse Model of Startle Disease

**DOI:** 10.3389/fnmol.2018.00167

**Published:** 2018-05-24

**Authors:** Natascha Schaefer, Fang Zheng, Johannes van Brederode, Alexandra Berger, Sophie Leacock, Hiromi Hirata, Christopher J. Paige, Robert J. Harvey, Christian Alzheimer, Carmen Villmann

**Affiliations:** ^1^Institute for Clinical Neurobiology, Julius-Maximilians-University of Würzburg, Würzburg, Germany; ^2^Institute of Physiology and Pathophysiology, Friedrich-Alexander-University Erlangen-Nürnberg, Erlangen, Germany; ^3^Princess Margaret Cancer Centre, University Health Network, University of Toronto, Toronto, ON, Canada; ^4^Research Department of Pharmacology, UCL School of Pharmacy, London, United Kingdom; ^5^Department of Chemistry and Biological Science, College of Science and Engineering, Aoyama Gakuin University, Sagamihara, Japan; ^6^School of Health and Sport Sciences, University of the Sunshine Coast, Sippy Downs, QLD, Australia; ^7^Sunshine Coast Health Institute, Birtinya, QLD, Australia

**Keywords:** glycine receptor, startle disease, β8-β9 loop, mouse model, fast decay, *shaky*

## Abstract

Mutations in GlyR α1 or β subunit genes in humans and rodents lead to severe startle disease characterized by rigidity, massive stiffness and excessive startle responses upon unexpected tactile or acoustic stimuli. The recently characterized startle disease mouse mutant *shaky* carries a missense mutation (Q177K) in the β8-β9 loop within the large extracellular N-terminal domain of the GlyR α1 subunit. This results in a disrupted hydrogen bond network around K177 and faster GlyR decay times. Symptoms in mice start at postnatal day 14 and increase until premature death of homozygous *shaky* mice around 4–6 weeks after birth. Here we investigate the *in vivo* functional effects of the Q177K mutation using behavioral analysis coupled to protein biochemistry and functional assays. Western blot analysis revealed GlyR α1 subunit expression in wild-type and *shaky* animals around postnatal day 7, a week before symptoms in mutant mice become obvious. Before 2 weeks of age, homozygous *shaky* mice appeared healthy and showed no changes in body weight. However, analysis of gait and hind-limb clasping revealed that motor coordination was already impaired. Motor coordination and the activity pattern at P28 improved significantly upon diazepam treatment, a pharmacotherapy used in human startle disease. To investigate whether functional deficits in glycinergic neurotransmission are present prior to phenotypic onset, we performed whole-cell recordings from hypoglossal motoneurons (HMs) in brain stem slices from wild-type and *shaky* mice at different postnatal stages. *Shaky* homozygotes showed a decline in mIPSC amplitude and frequency at P9-P13, progressing to significant reductions in mIPSC amplitude and decay time at P18-24 compared to wild-type littermates. Extrasynaptic GlyRs recorded by bath-application of glycine also revealed reduced current amplitudes in *shaky* mice compared to wild-type neurons, suggesting that presynaptic GlyR function is also impaired. Thus, a distinct, but behaviorally ineffective impairment of glycinergic synapses precedes the symptoms onset in *shaky* mice. These findings extend our current knowledge on startle disease in the *shaky* mouse model in that they demonstrate how the progression of GlyR dysfunction causes, with a delay of about 1 week, the appearance of disease symptoms.

## Introduction

Glycinergic inhibition is prominent in brain stem and spinal cord, where it is involved in essential processes such as motor control (Lynch, 2004), inflammatory pain sensitization (Harvey et al., 2004) and rhythmic breathing (Manzke et al., 2010). GlyR defects have also been implicated in startle disease (Harvey et al., 2008) autism spectrum disorder (Pilorge et al., 2015; Zhang et al., 2017) and panic disorders (Deckert et al., 2017). Glycine receptors (GlyRs) are integrated into the nerve-muscle circuit, where they are postsynaptically expressed in the membrane of motoneurons. Upon glycine-release from neighboring inhibitory interneurons, GlyRs are activated and chloride ion flux leads to hyperpolarization of the motoneurons, regulating excitation of the motoneurones and thus controlling muscle contraction (Schaefer et al., 2012). Defects in glycinergic transmission are the underlying cause of the neurological motor disorder hyperekplexia (OMIM 149400, startle disease, stiff baby syndrome). Human hyperekplexia is caused by mutations in the *GLRA1, GLRB*, or *SLC6A5* genes, encoding GlyR α1 and β subunits and the glycine transporter GlyT2. Symptoms in human hyperekplexia range from exaggerated startle reactions - due to unexpected acoustic or tactile stimuli - to muscle stiffness, apnea, and loss of postural control. Individuals with startle disease are typically treated with low doses of the benzodiazepine clonazepam, a positive allosteric modulator of GABA_A_ receptors (Christian et al., 2013).

Mice carrying *Glra1* (*spasmodic, oscillator, shaky, nmf11, cincinnati*) or *Glrb* (*spastic*) mutations have served as models of startle disease. However, it is noteworthy that disease symptoms are typically severe in most mouse startle disease models. Phenotypic symptoms typically start at day P14 and increase during the second and third week of life until premature death between weeks 4 and 6. *Spasmodic* mice harbor the missense mutation A52S in the GlyR α1 subunit A loop, leading to decreased ligand affinity and potency (Ryan et al., 1994). Homozygous *cincinnati* and *oscillator* mice represent GlyR α1 subunit null mutations, the former caused by duplication of *Glra1* exon 5, the latter due to a microdeletion in exon 8, both resulting in mRNA mis-splicing and truncated non-functional GlyR α1 subunits (Buckwalter et al., 1994; Holland et al., 2006). *Spastic* mice result from aberrant splicing of the GlyR β subunit mRNA, due to a LINE-1 element insertion in intron 6 (Becker et al., 2012). *Nmf11* harbors a GlyR α1 subunit N46K missense mutation, resulting in a reduction in the potency of the transmitter glycine and rapid deactivation mutant GlyR currents (Wilkins et al., 2016). Lastly, the recently characterized spontaneous mouse mutant *shaky* carries a missense mutation Q177K in the β8-β9 loop of the GlyR α1 subunit extracellular domain (Schaefer et al., 2017). Few of these mutants have been studied using *ex vivo* electrophysiological approaches. Recordings from hypoglossal motoneurons of adult *oscillator* or *spastic* mice have revealed reduced amplitude and frequency of mIPSCs (Graham et al., 2003, 2006). GlyR defects have also been studied in zebrafish, where some GlyR genes have undergone duplication during evolution (Hirata et al., 2005). In this model organism, mutation of the GlyR β subunit gene (Hirata et al., 2005) or morpholino knockdown of the GlyR α1 or α4a subunits also leads to defective startle and escape responses (Ganser et al., 2013; Leacock et al., 2018).

GlyRs belong to the superfamily of Cys-loop receptors (CLRs) and are pentameric receptor complexes. At synapses, these pentameric receptors are composed of α- and β- subunits anchored via the scaffolding protein gephyrin. Various ion channel stoichiometries, e.g., 3α:2β or 2α:3β subunits have been described (Yang et al., 2012; Patrizio et al., 2017). The large extracellular domains (ECDs) form ligand-binding sites at the interface between adjacent subunits, which are constituted by loops A-C from one subunit (principle subunit refers to (+) site) and loops D-F (loop F is further referred to as loop β8-β9, (–) site refers to complementary subunit) from the neighboring subunit (Hibbs and Gouaux, 2011). Several *in vitro* studies have revealed that GlyR function is highly dependent on ECD loop structures of the receptor, e.g., phenylalanine 159, which is localized in loop B was shown to contribute to cation-π interaction with the incoming ligand. This process is essential prior to channel opening (Pless et al., 2011). Moreover, loop C plays a role in transmitting the activation signal to the rest of the channel. This loop undergoes large rearrangements upon ligand binding, which is further translated to transmembrane (TM) domains (Althoff et al., 2014). Loop β8-β9 has been suggested to play a major role in linking ligand binding to channel opening. The published structural model for GlyR α1 showed a coupling of movements within the ECDs, including the β8-β9 loop, proceeding to the TM helices resulting in their tilting and enabling ion channel opening and closing (Du et al., 2015; Huang et al., 2015). The *shaky* mutation, Q177K, localized in the β8-β9 loop represents the first *in vivo* model where this ECD loop structure has been disrupted. Affected mice show largely impaired glycinergic function, which is in line with the known structural importance of the β8-β9 loop.

The GlyR α1β subtype is the most abundant adult isoform in spinal cord and brain stem nuclei. It is known, that all GlyR subunits undergo developmental regulation, with α1 and β subunit expression increasing from birth to P19 in spinal cord and brain stem nuclei (e.g., hypoglossal nuclei, pre-Bötzinger neurons). By contrast, α2 and α3 subunits decrease after a first peak at postnatal day 7–10 (Liu and Wong-Riley, 2013). Variation in developmental regulation of GlyR subunit expression between humans and mice account for differences in disease onset and progression. In humans, startle disease symptoms are evident during the first week of life, whereas in rodents symptoms are first observed during the second postnatal week around P14 (Ryan et al., 1992; Buckwalter et al., 1994). However, since GlyR gene expression is already evident in mice by P7, it is possible that functional deficits are already present within the second postnatal week before the onset of severe disease symptoms.

Here, we explored the importance of the *shaky* Q177K mutation focusing on the onset and progression of behavioral symptoms and functional GlyR deficits. Our data show that the Q177K mutation in the GlyR α1 subunit β8-β9 loop impairs motor coordination in mice and in zebrafish. GlyR α1 subunit expression starts 7 days prior to onset of the startle phenotype in mice. This is paralleled by functional deficits in glycinergic neurotransmission that become obvious at P9 and increase during disease progression. In conclusion, functional defects at the molecular level are present days before the disease phenotype is evident.

## Materials and methods

### Mouse lines

The *shaky* mutant mouse strain arose as a spontaneous mutation in the animal colony of C. Paige (University Health Network Research, Toronto, Canada) in a mixed 129X1/SvJ / C57BL6 strain. Mice were transferred into the animal facility of the Institute for Clinical Neurobiology (Würzburg, Germany), where mice were housed under pathogen-free conditions; water and food were available *ad libitum*. Experiments were approved by the local veterinary authority (Veterinaeramt der Stadt Wuerzburg), the Ethics Committee of Animal Experiments, i.e., Regierung von Unterfranken, Würzburg (License number 55.2-2531.01-09/14) and the University Health Network's Institutional Animal Facility. *Spasmodic* and *oscillator* mice were a gift from C.-M. Becker (Institute of Biochemistry, Friedrich-Alexander-University Erlangen-Nürnberg, Germany).

### Behavioral analysis

The neuromotor phenotype of homozygous *Glra1*^*sh*/*sh*^ mice was investigated by overall visual examination of the activity pattern including: hind-feet clasping, righting ability, time spent on back, time spent upright, resting, grooming, eating, assessment of gait by footprint recordings, body weight. Videos were recorded with the multi conditioning System from TSE (256060 series, Bad Homburg, Germany). *Video 1*: Impaired righting behavior and typical hind limb clasping at the onset of symptoms at P14. *Video 2*: Severe neuromotor phenotype at P22 with exaggerated startle response accompanied by rigidity of extremities and the back upon touching. The animals were monitored with the TSE MCS FCS – SQ MED software. Body weight was checked over a period of 6 weeks every 2–3 days. Diazepam was injected intraperitoneally at a concentration of 0.5 mg/kg in a total volume of 100 μl of sterile PBS. Control animals were injected with PBS.

### GlyR α1 subunit transcript analysis

Total RNA was isolated from spinal cord of a 3-week old *shaky* mutant and littermate controls using Trizol reagent (Gibco/ThermoFisher Scientific, Waltham, Massachusetts, USA). cDNA synthesis was performed using Superscript II™ (Invitrogen, Carlsbad, California, USA). RT-PCR reaction mixes for β-actin and GlyR α1 subunit exons 1–9 contained 1x PCR buffer, 1.5 mM MgCl_2_, 0.2 mM of each dNTP, 10 pmol of each primer (β-actin for: 5′-TCCCTGGAGAAGAGCTACGA-3′, rev: 5′-ATCTGCTGGAAGGTGGACAG-3′; GlyRex1 for: 5′-CAGCACTAGAATCTGGAAGATG-3′, GlyRex9 rev: 5′-CCATAGGCAGAGAAGTTGAAG-3′) and 1 U of Platinum Taq DNA Polymerase (Invitrogen, Carlsbad, California, USA). The PCR program consisted of 95°C 5 min followed by 32 × 95°C 30 s, 59°C 30 s and 72°C 1 min.

### PCR genotyping *Shaky* mice

Biopsies were taken from earmarks and digested overnight in 800 μl of TENS buffer (50 mM Tris, pH 8.0, 100 mM EDTA, 100 mM NaCl, 1% (w/v) SDS, 0.5 mg/ml proteinase K) at 55°C. The genomic DNA was extracted via isopropanol precipitation. For *shaky* mice a 185 bp fragment was amplified, covering the entire exon 6. PCR reactions were set up as follows: 1.25 mM dNTPs, 25 mM MgCl_2_, 5x GoGreen buffer, 1 U GoTaq polymerase 5 pmol/μl primers contained 10 pmol of each primer (Forward: 5′CTGAGTTCTCGCTGACCGAGC3′ Reverse: 5′CACCTGTGTTGTAGTGCTTG3′). A PCR program of 5 min 95°C, 32 × 20 s 95°C, 20 s 59°C and 20 s 72°C was used. The product was subsequently digested with the restriction endonuclease HpyCH4V (New England Biolabs, Ipswich, Massachusetts, USA), which is only able to cut wild-type and not *shaky* PCR product. For analysis, all PCRs were loaded on 2–4% agarose gels.

### Membrane preparation

For membrane protein analysis, crude cell membranes were prepared from mouse tissues (Sontheimer et al., 1989).

### Western blot

For SDS-PAGE, 11% polyacrylamide gels were freshly prepared, followed by Western blot on nitrocellulose membranes (GE Healthcare, Little Chalfont, Great Britain). Membranes were blocked for 1 h with 5% BSA in TBS-T (TBS with 1% Tween 20). Primary antibodies were incubated overnight at 4°C. GlyR proteins were detected with the GlyR α1 specific antibody mAb2b (cat. no. 146003 1:1,500, Synaptic Systems, Göttingen, Germany). β-Actin (cat. no. GTX26276, WB 1:5,000, GeneTex/Biozol, Eching, Germany) served as loading control. Signals were detected using the ECL plus system (GE Healthcare, Little Chalfont, Great Britain).

### Brain homogenates

*Glra1*^+/+^ and *Glra1*^*sh*/*sh*^ mice were sacrificed at 3–4 weeks of age. GlyR α1 Western blot analysis of combined spinal cord and whole brain samples was performed as previously described (Traka et al., 2006), using a polyclonal antibody against the N-terminus of the 48 kDa GlyR α1 subunit (dilution 1:200, Merck Millipore, Darmstadt, Deutschland). β-actin (cat. no. GTX26276, WB 1:5,000, GeneTex/Biozol, Eching, Germany) served as loading control. Data analysis of Western blots: The image quantification was performed using the ImageJ software (1.51)/Fiji (Schindelin et al., 2015). The data were analyzed using Student's *t*-test (analysis of variance) or one-way *ANOVA*, and values below *p* < 0.05 were considered significant. The values are displayed as means ± standard deviation (±SD).

### Zebrafish assay

Zebrafish were bred and assayed according to the guidelines set forth by Aoyama Gakuin University. Antisense morpholino oligonucleotides that target *Dhx37* (MO2-dhx37: 5′-ATCAAGTGTTTTACCTTGTTGCGGA-3′) were coinjected with or without zebrafish wild-type or mutant GlyRα1 RNA into zebrafish embryos at 1–2 cell stage (Hirata et al., 2013). Larval behaviors were observed at 48 h post-fertilization under a stereo microscope Leica MZ16F. Tactile stimuli were delivered to the tail using a pair of forceps. Responses of larvae were classified as follows: normal (a lateral turn and subsequent swimming), mildly affected (a dorsal bend followed by swimming of more than 2 cm), severely affected (a dorsal bend without escape swimming) according to a previous report (Hirata et al., 2013).

### Counting of motoneurons

To count motoneurons from the brain stem, mice were deeply anesthetized and transcardially perfused. Brain slices were stained with cresyl violet and motoneurons were counted in facial nerve in 15–16 sections of every tenth section of the brain stem. The raw counts were corrected for double counting of split nucleoli as described (Masu et al., 1993). Differences between groups were evaluated with Student's *t*-test (unpaired, significance level ^*^p < 0.05). The Graphics Prism Program (Graph Pad Software Inc., San Diego, California, USA) was used for calculation and data presentation.

### Brain stem slice preparation and whole-cell recordings

Electrophysiological experiments were performed on brain stem slices from 9 to 24 day old mice. After anesthesia and decapitation, brain stems were rapidly removed and immersed in ice-cold ‘high sucrose’ artificial cerebrospinal fluid (aCSF) containing (in mM): 75 sucrose, 125 NaCl, 3 KCl, 0.3 CaCl_2_, 7 MgCl_2_, 1.25 NaH_2_PO_4_, 25 NaHCO_3_, 30 D-glucose and bubbled with carbogen (95% O_2_/5% CO_2_, pH 7.4). Transverse slices 250 μm thick containing the hypoglossal motor nucleus (XIIn) were cut, transferred to warmed (35°C) high-sucrose aCSF for 10 min and kept thereafter in normal aCSF (see below) at room temperature for at least 1 h before being transferred individually to a submerged recording chamber, which was perfused with normal aCSF of the following composition (in mM) 125 NaCl, 3 KCl, 1.5 CaCl_2_, 1 MgCl_2_, 1.25 NaH_2_PO_4_, 25 NaHCO_3_ and 30 D-glucose at 30°C, gassed with 95% O_2_/5% CO_2_ (pH 7.4). Whole-cell recordings from neurons in hypoglossal motor nucleus were performed with patch pipettes filled with internal solution composed of (in mM): 130 CsCl, 3 MgCl_2_, 5 EGTA, 5 Hepes, 2 Na_2_-ATP, 0.3 Na_3_-GTP, and 5 QX-314 (pH 7.3). The electrode resistance ranged from 3 to 5 MΩ when filled with internal solution. Whole-cell currents were recorded at a holding potential of −70 mV (corrected for liquid junction potential), filtered (2 kHz) and sampled at 20 kHz using a Multiclamp 700B amplifier in conjunction with Digidata 1440A interface and pClamp10 software (Molecular Devices, Silicon Valley, California, USA).

Miniature glycinergic IPSCs (mIPSCs) were pharmacologically isolated by perfusing slices with aCSF containing the ionotropic glutamate receptor antagonist kynurenic acid (KA, 2 mM), GABA_A_ receptor antagonist bicuculline methiodide (BIC, 20 μM) and tetrodotoxin (TTX, 1 μM). Individual events were detected with Clampfit software (Molecular Devices, Silicon Valley, California, USA) using a template method with amplitude threshold set to 5–6^*^ σ_noise_. Peak amplitude, 10–90% rise time and 90–10% decay time were measured and averaged over a minimum of 20 events. For mIPSC kinetics only non-overlapping events with relatively fast rise times (<2 ms) and a smooth decay were included in the analysis. For dose-response curves, glycine (10–1,000 μM) was bath applied in the presence of TTX (1 μM), KA (2 mM) and BIC (10 μM). The peak current at a given concentration was averaged from values measured in individual neurons, plotted against glycine concentration, and fitted with a sigmoidal function for determination of EC_50_. Stationary noise analysis (SNA) was performed on glycine-evoked current responses with the help of the analysis software WinEDRv.3.5 (John Dempster, Strathclyde Software, University of Strathclyde, Glasgow, Great Britain) using the recorded DC current signal and a band-pass filtered AC-coupled version of this signal. Mean current (I_m_) and current variance (σ^2^) were computed from the DC and AC traces, respectively, for records where the whole-cell current was slowly changing due to glycine application. After subtraction of the control background variance, we fitted the variance-mean curve with a parabolic function. Estimates of the single channel current I_u_ were obtained from the number of channels (N_c_) and the open probability (P_o_) according to: σ^2^ = ${\text{I}}_{\text{u}}^{2}$N_c_P_o_(1-P_o_) (1) and I_m_ = I_u_N_c_P_o_ (2), which combine to give the parabolic function: σ^2^ (I_m_) = I_u_ I_m_–I_m_ 2/N_c_ (3). In some cases we were not able to get a satisfactory parabolic fit of the variance-mean relationship and we fitted only the initial portion of the graph with a linear relationship of the form: I_u_ = σ^2^/I_m_ (4). Channel-gating kinetics were measured from individual power spectra taken from the steady-state portion of the glycine-induced current. The one-sided net spectrum (for frequencies < 200 Hz) was fit with a single Lorentzian function and the time constant τ was calculated from the corner frequency fc according to: τ = 1/ 2π f_c_. In some cases, a better fit of the spectra was obtained using two Lorentzians and a single weighted time constant τ_w_ was calculated from the two time constants (τ_1_ and τ_2_) according to: τ_w_ = τ_1_ [S_o1_/(S_o1_ + S_o2_)] + τ_2_ [S_o2_/(S_o1_ + S_o2_)] (5) where S_o1_ and S_o2_ are the zero frequency spectral power for the two Lorentzian functions.

## Results

Our current view of startle disease focusses on GlyR and GlyT2 variants either affecting receptor/transporter function or biogenesis. However, *in vivo* compensatory mechanisms are still a matter of debate. Using the spontaneous mouse GlyR α1 subunit mutant *shaky*, it has been demonstrated that the extracellular β8-β9 loop is a key structural and functional element for GlyR signaling that influences conformational changes including the M3-M4 domain involved in synaptic clustering and the formation of the glycine-binding pocket (Schaefer et al., 2017). Here, we give a detailed genetic, behavioral and electrophysiological account of this mouse model and investigate temporal differences between disease onset, GlyR expression and GlyR function.

**Figure 1:**
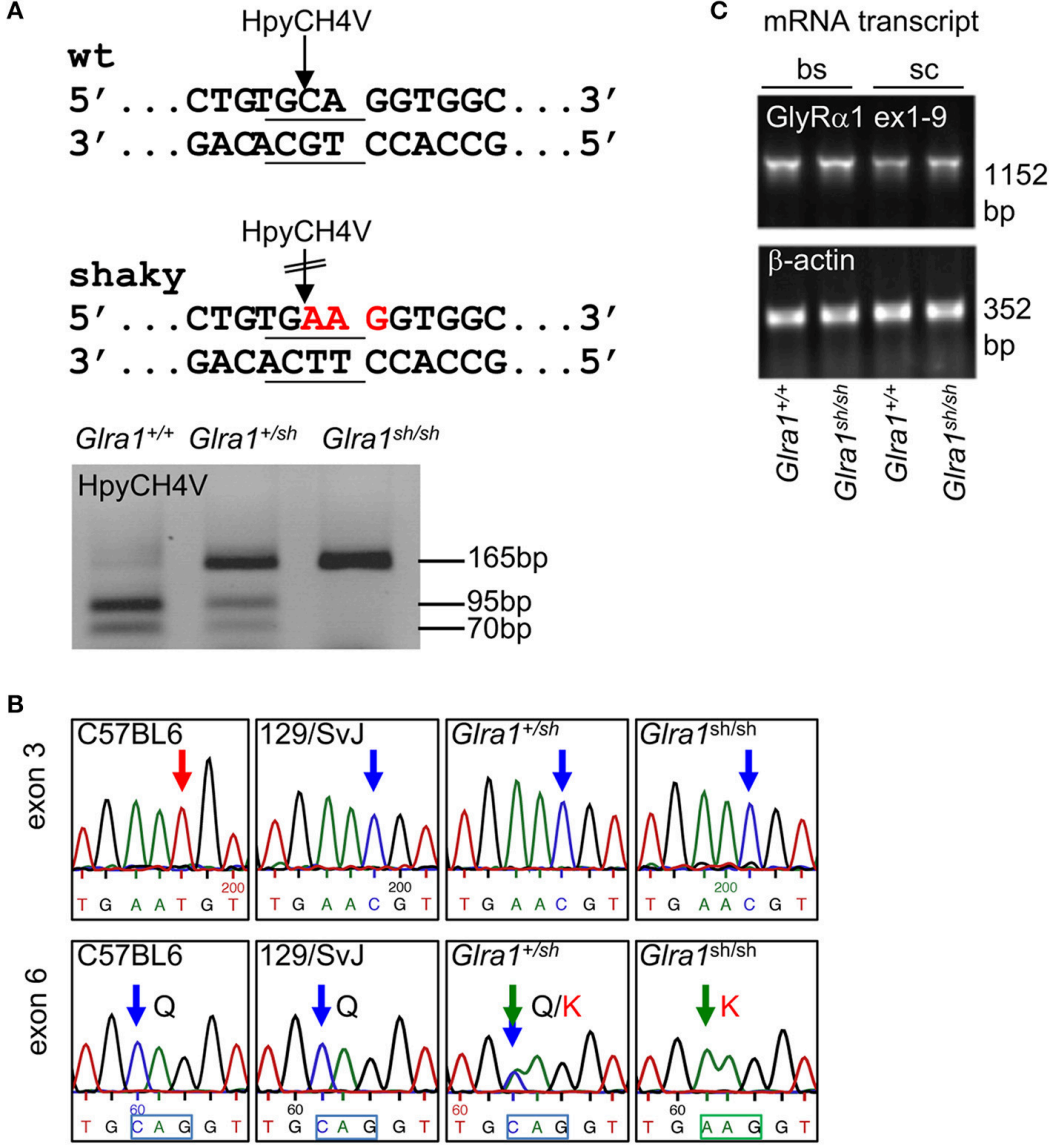
Genotype of *shaky* mice. **(A)** The recognition site for the restriction enzyme HpyCH4V is disrupted by the *shaky* mouse mutation in exon 6; left panel. PCR genotyping of *shaky* (*Glra1*^*sh*/*sh*^), wild-type (*Glra1*^+/+^) and heterozygous (*Glra1*^+/*sh*^) mice with subsequent HpyCH4V digest (right panel). **(B)** Sequencing chromatograms of wild-type strains C57BL6 and 129/SvJ, heterozygous *Glra1*^+/*sh*^, and homozygous *Glra1*^*sh*/*sh*^ showing a c.T198C transition in exon 3 and a c.C613A transition in exon 6. Both wild-type mouse strains are shown since the *shaky* mutation arose in a hybrid background of C57BL6 and 129/SvJ. **(C)** RT-PCR analysis of GlyR α1 subunit mRNA levels in spinal cord (sc) and brain stem (bs) of wild-type (*n* = 4) and *shaky* mice (*n* = 4). β-actin cDNA was amplified as a reference gene to ensure equal cDNA content in all samples.

The novel murine *shaky* mutation (GlyR α1^Q177K^) arose as a spontaneous mutation in a hybrid background of C57BL6 and 129SvJ (Schaefer et al., 2017). A rapid differentiation between mouse genotypes was enabled by the disruption of a HpyCH4V restriction site (Figure 1A). Sequencing of *Glra1* from homozygous *shaky* mice (*Glra1*^*sh*/*sh*^) and littermate controls revealed two transitions: c.T198C in exon 3 (synonymous, p.N38N) and c.C613A in exon 6 (missense, p.Q177K, numbers refer to mature protein) (Figure 1B). The exon 3 transition is due to to a single nucleotide variation in the background mouse lines C57BL6 and 129SvJ of the *shaky* origin (Figure 1B). Although synonymous at the protein level, nucleotide sequence variations might disrupt or result in exonic splicing enhancer (ESE) sites and thus influence splicing events of the affected mRNA (Becker et al., 2012).

To exclude aberrant splicing, GlyR α1 subunit mRNA was analyzed for possible ESE sites at sequence variations in exon 3 and exon 6 observed in *shaky* mice. The sequence of exon 3 harboring the c.T198C transition in exon 3 did not reveal an ESE site in the wild-type or in the mutated sequence. The sequence transition in exon 6 corresponded to an ESE site for the splice factor SRSF1 present in the wild-type but not in the mutant sequence (http://krainer01.cshl.edu/cgi-bin/tools/ESE3/esefinder.cgi?process=home). This potential ESE site in the GlyR α1 nucleotide sequence ^611^TGCAGGT^617^ had a score of 2.23402, threshold: 1.956. However, we did not observe aberrant splicing of GlyR α1 subunit mRNA (Figure 1C) in either wild-type (*Glra1*^+/+^) or homozygous *shaky* (*Glra1*^*sh*/*sh*^) mice.

**Figure 2:**
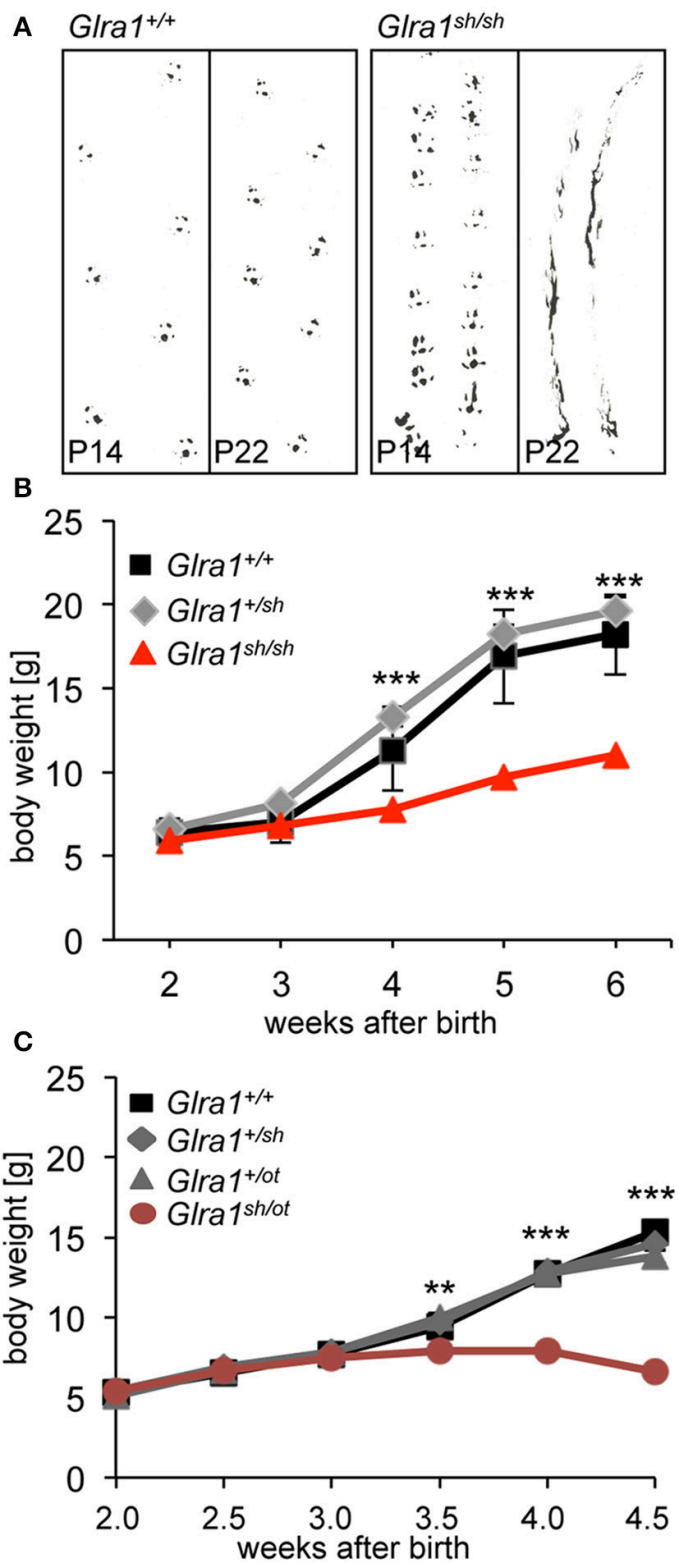
The neuromotor phenotype of *shaky* mice is due to a glycinergic defect. **(A)** Footprint recordings. Mice painted on their hind paws were placed on a sheet of paper in a 30 cm tunnel. Representative *Glra1*^+/+^ and *Glra1*^*sh*/*sh*^ footprints are shown at P14 and P22. **(B)** Homozygous *Glra1*^*sh*/*sh*^ mice (*n* = 9) gain less body weight than wild-type (*n* = 4) and heterozygous littermates (*n* = 19). Survival curves of *Glra1*^*sh*/*sh*^, *Glra1*^+/*sh*^, *Glra1*^+/+^ mice. *Glra1*^*sh*/*sh*^ mice die between weeks 3 and 6 of life. **(C)** Loss in body weight of heterozygous *Glra1*^*sh*/*ot*^ mice following onset of symptoms (P14), *Glra1*^+/+^ *n* = 19, *Glra1*^+/*sh*^ *n* = 9, *Glra1*^+/*ot*^ *n* = 25, *Glra1*^*sh*/*ot*^ *n* = 10, *ANOVA* ^**^p < 0.01, ^***^p < 0.001.

The phenotype of homozygous *shaky* mice becomes apparent at the age of 2 weeks, at the time point when GlyRs containing the α2 subunit are switched for adult GlyR α1β isoforms in the spinal cord and brain stem. A severe motor defect characterized by tremor, muscle spasms, twitchy tail, stiffness, and poor motor control compared to age-matched littermates becomes evident. Homozygous *shaky* mice can be easily recognized by typical hind feet clasping when picked up by their tails, and abnormal gait with uneasy footsteps and skidding of hind limbs during tremor episodes (Figure 2A). These neuromotor symptoms are similar to *oscillator* mice (Buckwalter et al., 1994), a mouse model for startle disease/hyperekplexia with a progressive severe phenotype. During episodes of tremor, *shaky* mice display a hunched, stiff posture and often end up on the tip of their toes, which causes them to fall over on their side or back (Videos 1, 2). Homozygous mutant mice are usually smaller than their littermates with a significant decrease in body weight observed after P28, 2 weeks after phenotypic onset (Figure 2B). On average, *shaky* mice die at the age of 3–6 weeks. A 100% overlap between genotyped homozygous *shaky* mice, the observed typical startle phenotype and death after 3–6 weeks was observed. Heterozygous *shaky* mice were bred to heterozygous *oscillator* and *spasmodic* mice. Homozygous *oscillator* mice die at the age of 3 weeks, homozygous *spasmodic* mice have a normal life span. Again with a delay to phenotypic symptoms, significantly lower body weight (P24-32) was determined for *Glra1*^*sh*/*ot*^ mice (Figure 2C). Backcross experiments of *shaky* animals into the *spasmodic* mouse line led to a mild phenotype and survival of *Glra1*^*sh*/*spd*^ animals similar to the homozygous *spasmodic* animals. No differences in body weight were observed for *Glra1*^*sh*/*spd*^ animals compared to wild-type controls or heterozygous *Glra1*^+/*sh*^ and *Glra1*^+/*spd*^ animals (data not shown). Due to the survival of *Glra1*^*sh*/*spd*^ animals, we analyzed the expression pattern of the GlyR α1 subunit during development. Expression of the GlyR α1 subunits was detected in spinal cord and brainstem samples from P0 to P28 in backcross experiments of the *shaky* line into the *spasmodic* line.

**Figure 3:**
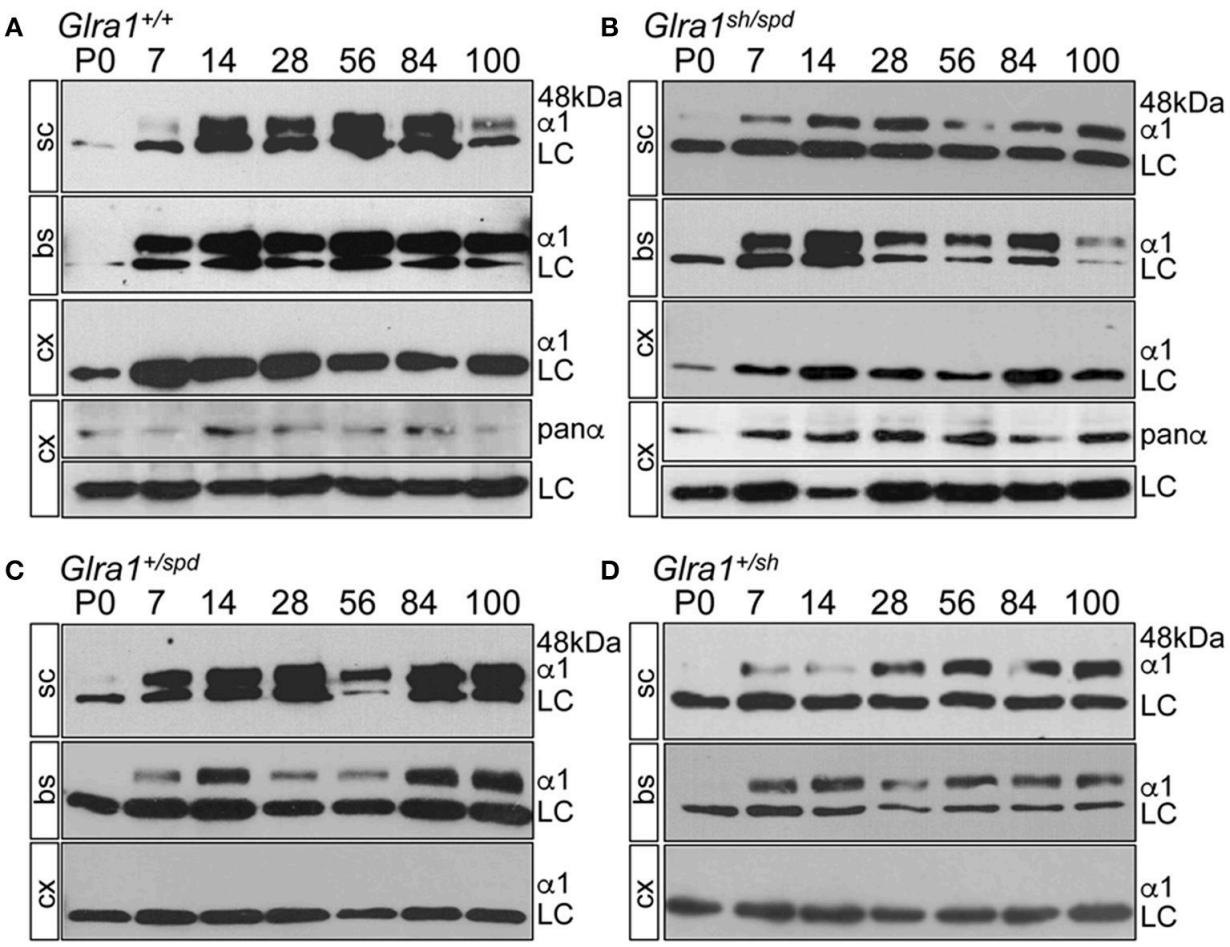
Backcross of *shaky* mouse line into the *spasmodic* mouse line. Developmental expression of the GlyR α1 subunit in *shaky* mice and after backcross into the mouse line *spasmodic* **(A–D)**. After backcross of *shaky* into the *spasmodic* line, the expression profile was determined in spinal cord (sc), brain stem (bs) and cortex (cx) for stages P0, P7, P14, P28, P56, P84, and P100. **(A)** *Glra1*^+/+^, **(B)** *Glra1*^*sh*/*spd*^, **(C)** heterozygous *Glra1*^+/*spd*^, and **(D)** heterozygous *Glra1*^+/*sh*^. Cortex (cx) served as negative control for GlyR α1. GlyR α1 subunit was stained with mAb2b (48 kDa), and β-Actin (46 kDa) served as a loading control (LC). Cortex samples were probed with a GlyR pan-α antibody (mAb4a) labeling other GlyR α subunits in the cortex **(A,B)**.

GlyR α1 subunit expression started at P7 in spinal cord and in brain stem of *Glra1*^*sh*/*spd*^ animals indistinguishable from *Glra1*^+/+^ mice (Figures 3A–D). No GlyR α1 subunit expression was observed in cerebral cortex (using a polyclonal antibody against the N-terminus of the 48 kDa GlyR α1 subunit (dilution 1:200, Merck Millipore, Darmstadt, Deutschland), which served as a negative control. This does not necessarily mean that other GlyR α subunits are also not expressed. Previously we have shown, using a pan-GlyR α subunit antibody, that other GlyR α subunits are expressed in the cortex of *Glra1*^+/+^, *Glra1*^+/*sh*^, and *Glra1*^*sh*/*sh*^ animals at P28 (Schaefer et al., 2017) although expression was decreased compared to brainstem and spinal cord controls. Here, the subunit switch to increased GlyR α1 subunit levels after birth was completed before onset of symptoms at P14. Despite a normal life span, *Glra1*^*spd*/*sh*^ mice developed of a startle phenotype. To determine if symptomatic onset influences GlyR α1 expression, the GlyR α1 protein was monitored up to P100 demonstrating constant GlyR α1 subunit levels between P14-P100 (Figure 3B). There were no obvious differences between symptomatic *Glra1*^*spd*/*sh*^ mice and non-symptomatic *Glra1*^+/*sh*^ and *Glra1*^+/*spd*^ animals.

**Figure 4:**
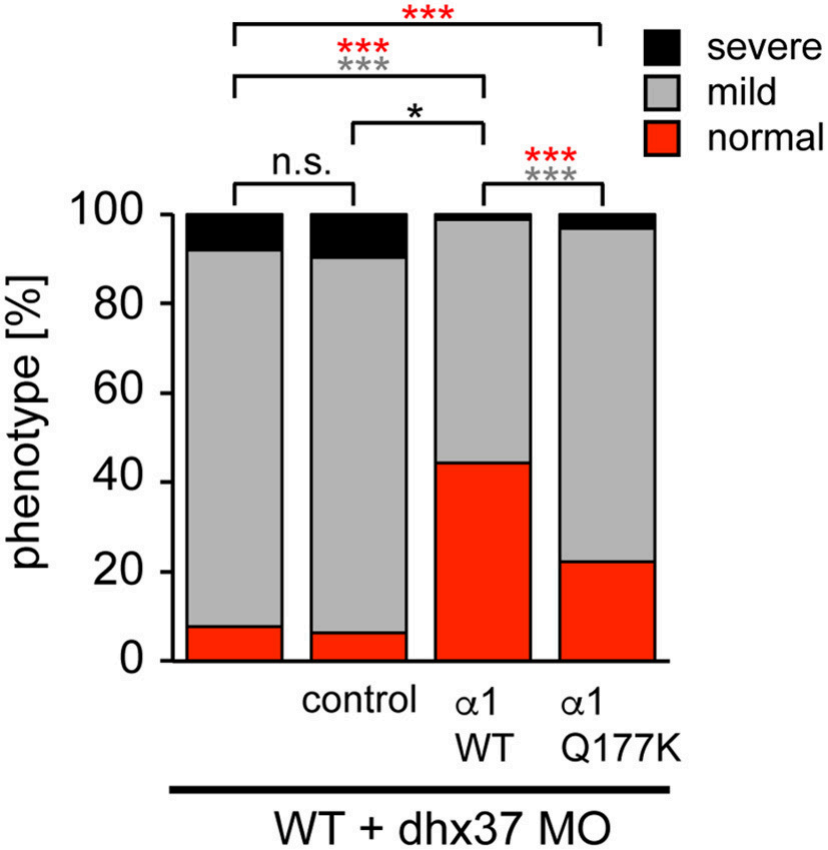
The Q177K mutation prevents functional recovery in the zebrafish mutant *Dhx37*. Stacked bar diagram representing four different conditions of morpholino or mRNA injections into zebrafish larvae. MO2-dhx37 is present in all four conditions. Second lane shows additional injection of a control mRNA, third line injection of wild-type GlyR α1 subunit mRNA and fourth lane injection of GlyR α1^Q177K^ mRNA. Black squares demonstrate the portion of severe escape behavior of zebrafish larvae in percent (%), gray squares display the portion of the mild phenotype, and red the normal escape behavior. Animals analyzed for control condition *n* = 31–38, animals for GlyR α1 wild-type *n* = 79, GlyR α1^Q177K^ *n* = 63. *P*-value represents significance level with ^*^p < 0.05 and ^***^p < 0.001.

Since the touch-evoked startle and escape behavior in zebrafish mirrors a startle phenotype in mice or humans, we used the *Dhx37* zebrafish model to analyze the GlyR α1^Q177K^ mutation. Normally, zebrafish embryos respond to tactile stimuli with escape contractions that typically consist of two-to-three rapid, alternating contractions of the axial muscles (Hirata et al., 2005). Knockdown of *Dhx37* decreases GlyR α subunit mRNAs (GlyR α1, α3, and α4a subunits) causing abnormal escape responses in zebrafish (Hirata et al., 2013). The three different types of escape behavior, normal escape behavior (a lateral turn and subsequent swimming), a mild version of abnormal escape behavior (a dorsal bend followed by swimming of more than 2 cm) and the severe change in escape behavior (a dorsal bend without escape swimming) were compared. Co-injection of GlyR α1 subunit wild-type RNAs with a morpholino against *Dhx37* (MO2-dhx37) increased the normal escape behavior compared to morpholino alone, while co-injection of GlyR α1^Q177K^ with MO2-dhx37 was not able to recover normal escape behavior to the same extent as wild-type GlyR α1 (Figure 4, Table 1). Co-injection of control RNAs with MO2-dhx37 did not ameliorate the escape response. These results demonstrate that the GlyR α1 subunit Q177K mutant is incapable of recovering disrupted motor phenotypes in zebrafish.

**Table 1:** Behavioral analysis of zebrafish larvae.

		**Normal**		**mild**	**severe**	**total number**	**normal (%)**	**mild (%)**	**severe (%)**	**total number (%)**		
wt + dhx37 MO		3		32	3	38	8	84	8	100		
wt + dhx37 MO + control RNA		2		26	3	31	6	84	10	100		
wt + dhx37 MO + GlyR α1		35		43	1	79	44	55	1	100		
wt + dhx37 MO + GlyR α1^Q177K^		14		47	2	63	22	75	3	100		
	**normal**				**mild**				**severe**			
	wt + dhx37 MO	wt + dhx37 MO + control RNA	wt + dhx37 MO + **GlyR α1**	wt + dhx37 MO + GlyR α1^Q177K^	wt + dhx37 MO	wt + dhx37 MO + control RNA	wt + dhx37 MO + GlyR α1	wt + dhx37 MO + GlyR α1^Q177K^	wt + dhx37 MO	wt + dhx37 MO + control RNA	wt + dhx37 MO + GlyR α1	wt + dhx37 MO + GlyR α1^Q177K^
wt + dhx37 MO		n.s.	^***^	^***^		n.s.	^***^	n.s.		n.s.	n.s.	n.s.
wt + dhx37 MO + control RNA	n.s.		^***^	^***^	n.s.		^***^	n.s.	n.s.		^*^	n.s.
wt + dhx37 MO + GlyR α1	^***^	^***^		^***^	^***^	^***^		^***^	n.s.	^*^		n.s.
wt + dhx37 MO + GlyR α1^Q177K^	^***^	^***^	^***^		n.s.	n.s.	^***^		n.s.	n.s.	n.s.	

* *p < 0.05*,

*** *p < 0.001, χ^2^ = quadrate test*.

**Figure 5:**
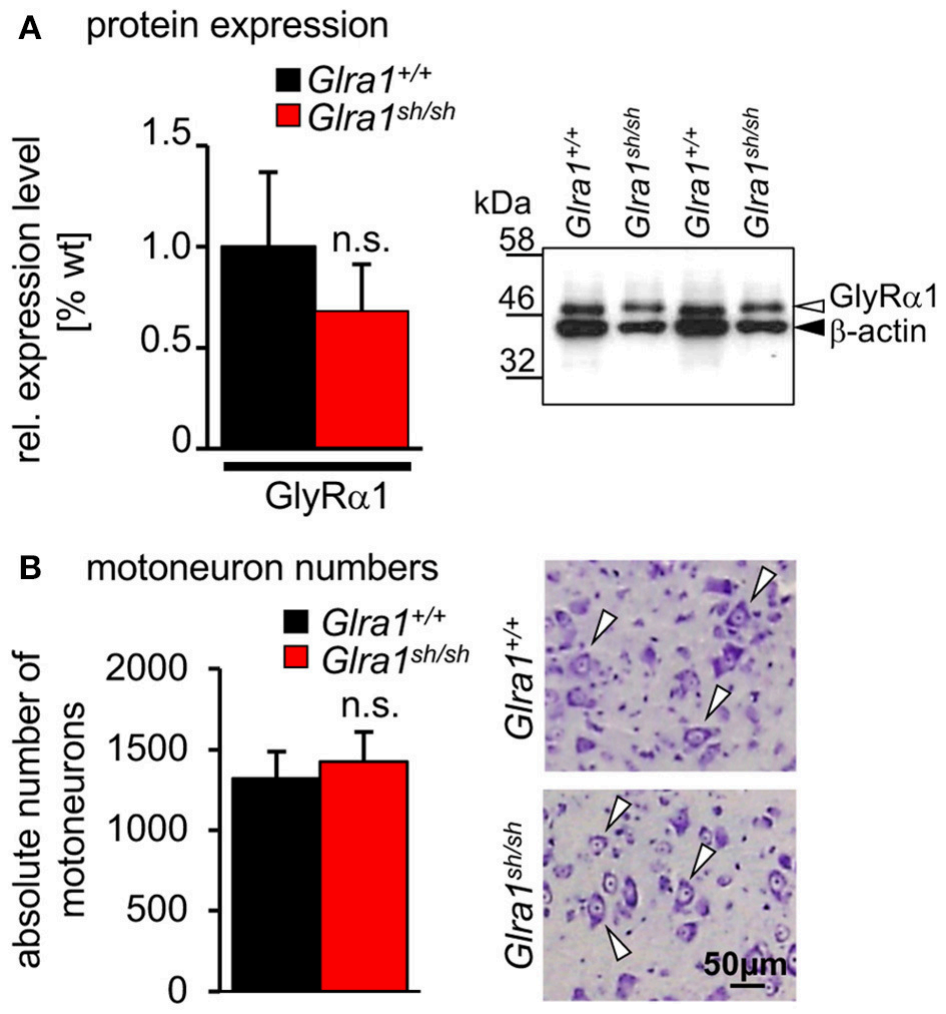
Expression level and number of motoneurons in wild-type and homozygous *shaky* mice. **(A)** Western blot analysis of GlyR α1 subunit protein expression in whole brain and spinal cord homogenates. Normalization with β-actin showed no significant differences of GlyR α1 protein levels between the two genotypes (*n* = 3), right image shows an example of the Western blot from two different animals of each genotype (*Glra1*^+/+^ and *Glra1*^*sh*/*sh*^). **(B)** Quantitative analysis of motoneuron numbers. The quantification was made using 3–4 animals of each genotype (*Glra1*^+/+^, *n* = 4; *Glra1*^*sh*/*sh*^, *n* = 3). Right images are examples of brain stem sections from *Glra1*^+/+^ and *Glra1*^*sh*/*sh*^ with arrow heads pointing to motoneurons. Error bars represent standard deviations (S.D.).

Biochemical analysis revealed no differences in the relative expression of GlyR α1 subunits in mixed whole-brain and spinal cord homogenisates of homozygous *shaky* mice in comparison to *Glra1*^+/+^ mice (*n* = 3 each genotype). The GlyR α1 amount was normalized to the control protein β-actin. The wild-type GlyR α1 value was set to 1 (Figure 5A). These data suggest that there are no significant differences of the GlyR α1 subunit in wild-type (relative expression 1.0 ± 0.37) vs. *shaky* mice (relative expression 0.63 ± 0.23) when combined brain and spinal cord samples were analyzed. We also examined the possibility that the phenotype in *shaky* mice was due to a loss of motoneurons in the brain stem, which could also be a cause for the observed symptoms. Motoneurons were counted in facial nerve in 15–16 sections of every tenth section of the brain stem. However, no differences in the number of motoneurons were detected in brain stem nuclei (number of motoneurons *Glra1*^+/+^ 1,317 ± 169 with *n* = 4, *Glra1*^*sh*/*sh*^ 1,424 ± 183 with *n* = 3) (Figure 5B), which is in line with previous data demonstrating similar numbers of spinal cord motoneurons in both genotypes (Schaefer et al., 2017).

**Figure 6:**
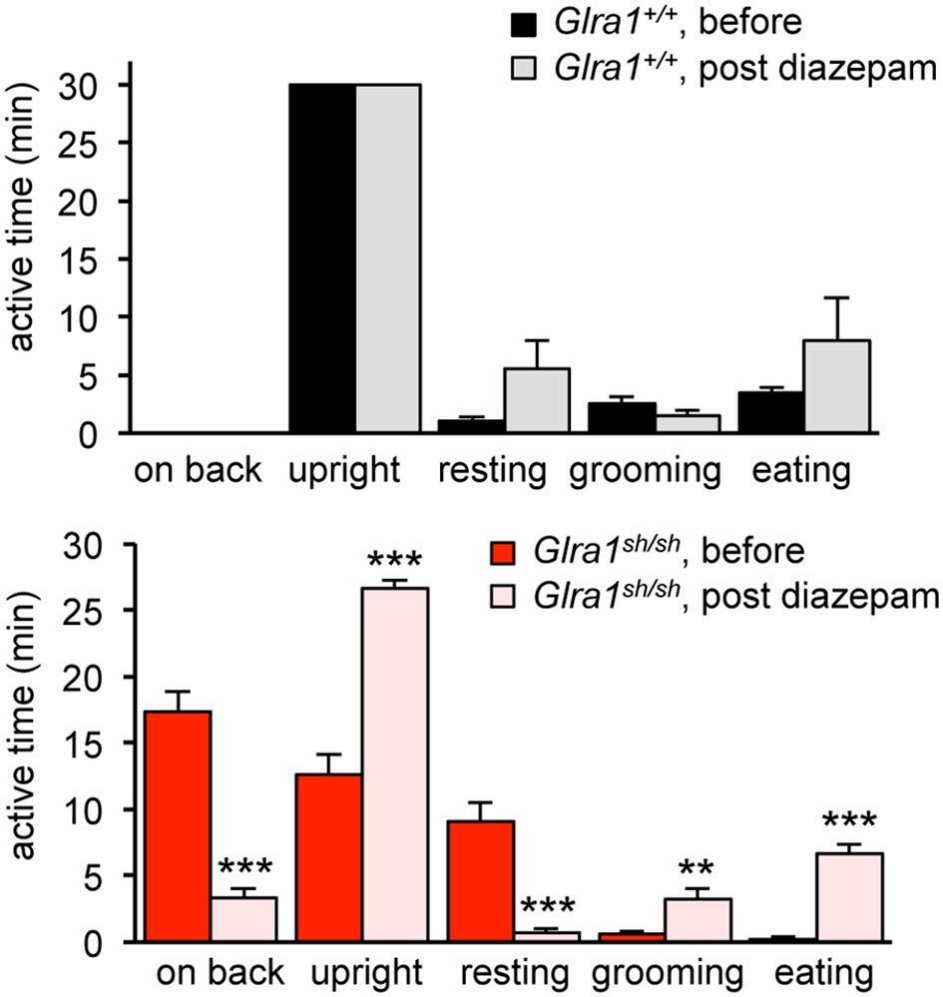
The neuromotor phenotype of *shaky* mice improves after diazepam treatment. Overall activity of *Glra1*^*sh*/*sh*^ (*n* = 8) mice before and after diazepam injection is displayed as time spent on a certain activity. The following activities within a 30 min time period were counted: falling (time spent on back/side vs. upright), resting (defined as the animal sitting in one spot for more than 10 s without any activity), rearing, grooming, eating/drinking, black panel *Glra1*^+/+^ (*n* = 4) and red panel *Glra1*^*sh*/*sh*^ mice. Level of significance ^**^p < 0.01 and ^***^p < 0.001.

Humans with startle disease are typically treated with diazepam, a positive allosteric modulator of GABA_A_ receptors. Therefore, *shaky* mice were injected with diazepam and the phenotype of affected mice was compared before and after treatment. Diazepam treatment indeed resulted in a decrease in phenotype severity, as evident by improved overall behavior and enhanced activity, e.g., time spending on back or in the upright position, resting, grooming, eating (Figure 6). The overall activities of *Glra1*^+/+^ and *Glra1*^*sh*/*sh*^ mice were counted 30 min before and after diazepam injection. While control mice were less active after diazepam treatment, likely due to the sedative nature of this drug (Figure 6), *shaky* mice were more active. Although *shaky* mice still lost balance after diazepam treatment, they were able to right themselves faster (before 13±2 min, after 28±0.3 min) after treatment and spent significantly less time on their sides and backs (before 17 ± 2 min, after 1.7 ± 0.3 min) (Figure 6). Resting (before 5.3 ± 1.3 min, after 1 ± 0.5 min), grooming (before 0.6 ± 0.3 min, after 4.7 ± 1.6 min), and time spent eating (before 0.3 ± 0.3 min, after 8.7 ± 1.3 min) also improved significantly upon treatment with diazepam. This argues for GABAergic compensation of glycinergic deficits but importantly demonstrates that GlyR mutant mice and wild-type mice respond differently to similar doses of diazepam.

**Figure 7:**
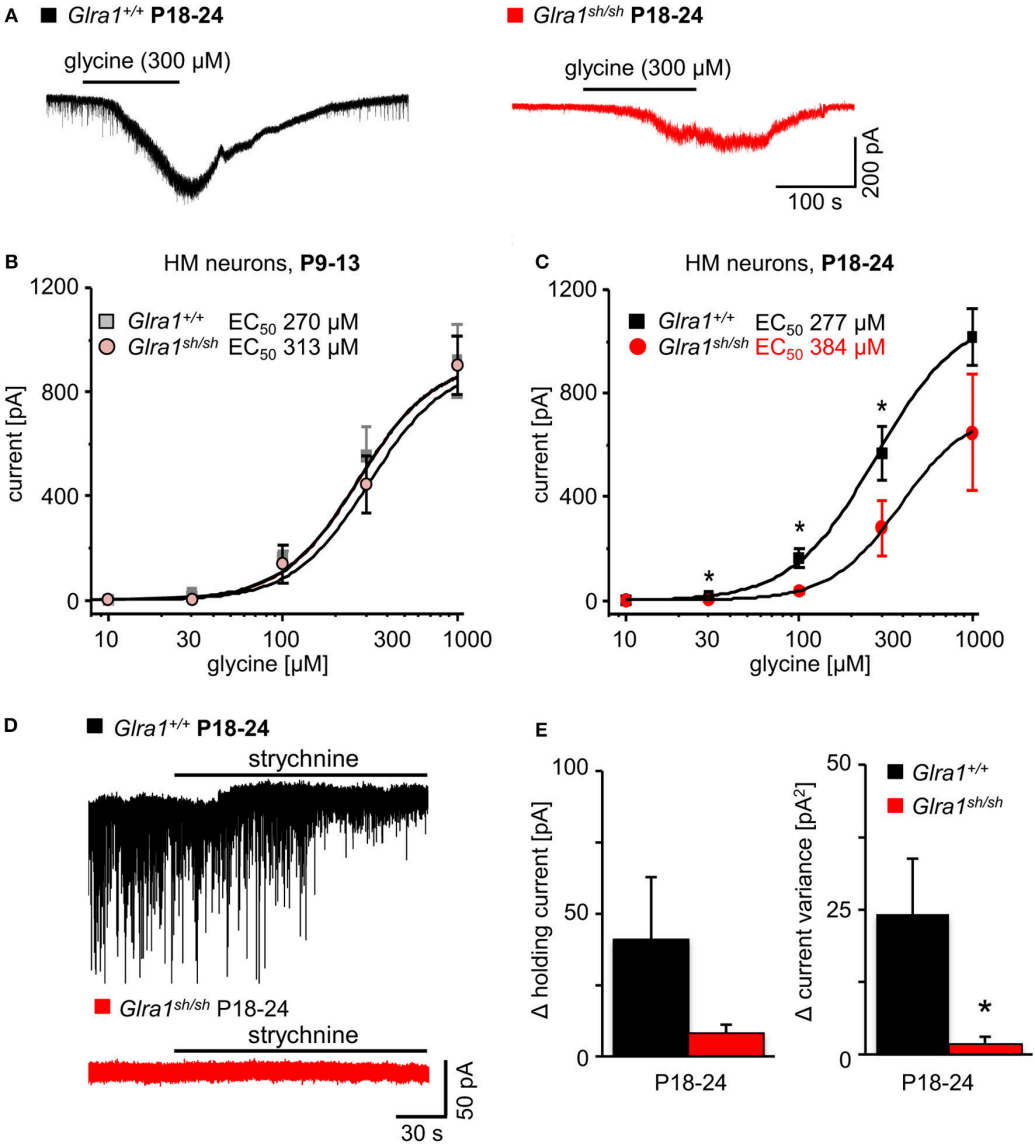
Developmental shift of glycinergic responses in *Glra1*^*sh*/*sh*^ hypoglossal motoneurons. Whole-cell voltage-clamp recordings were obtained from hypoglossal motoneurons (HMs) held at −70 mV, with symmetrical Cl^−^ concentrations inside and outside the cell. **(A)** Representative traces illustrate reduced glycine current in a *Glra1*^*sh*/*sh*^ HM (red) when compared to a *Glra1*^+/+^ HM (black), both from P18-24 mice. **(B)** At an earlier postnatal stage (P9-13), dose-response curves for glycine-induced currents showed no difference between HMs from *Glra1*^+/+^ (*n* = 7) and *Glra1*^*sh*/*sh*^ mice (*n* = 8). **(C)** Dose-response curves of *Glra1*^+/+^ (*n* = 6–11) and *Glra1*^*sh*/*sh*^ (*n* = 8–11) HMs from more mature mice (P18-24) with severe symptoms displayed significant divergence. The EC_50_ of glycine was significantly increased in *Glra1*^*sh*/*sh*^ mice. **(D)** Typical traces from P18-24 HMs illustrate lack of tonic, strychnine (2 μM)-sensitive glycine current in mutant HMs. **(E)** Bar diagrams summarize loss of glycinergic tone in HMs of *Glra1*^*sh*/*sh*^ mice, as indicated by missing changes in holding current (left columns) and current variance (right columns) upon application of strychnine. *P*-value of significance ^*^p < 0.05.

**Figure 8:**
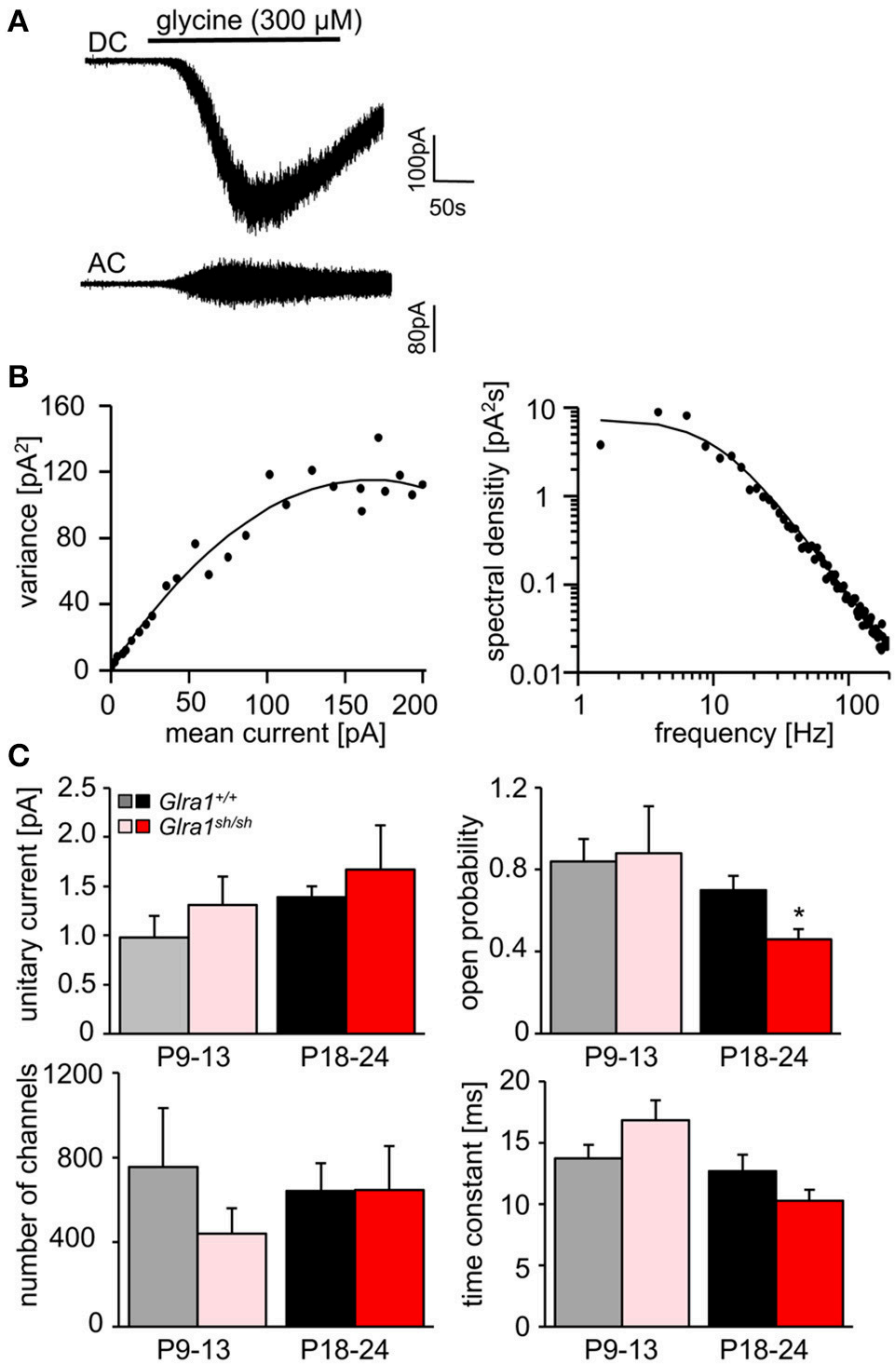
Stationary noise analysis of glycine-evoked whole-cell currents in wild-type and mutant HMs. **(A)** Example of a DC whole-cell current response (top) to glycine in a *Glra1*^+/+^ HM (P18-24) and the corresponding AC-coupled signal (bottom). **(B)** Variance-mean plot where AC current noise was plotted against DC current for the traces in **(A)**. The variance (σ^2^) vs. mean current (I) relationship was fitted with a parabolic function of the form: σ^2^ = *iI*− *I*^2^/*N* to obtain the unitary current (i) and the number of channels open (N) at the peak of the current response to glycine. Power spectrum of the glycine-evoked current noise in **(A)** was fit with a single Lorentzian function, and the time constant was calculated from the cut-off frequency f_c_ according to: τ = 1/2π*f*_c_. Points above 200 Hz have been omitted, right panel in **(B)**. **(C)** Comparisons of unitary currents (P9-13 *Glra1*^+/+^ *n* = 7, *Glra1*^*sh*/*sh*^ *n* = 5; P18-24 *Glra1*^+/+^ *n* = 9, *Glra1*^*sh*/*sh*^ *n* = 7), channel open probability (P_open_) (P9-13 *Glra1*^+/+^ *n* = 4, *Glra1*^*sh*/*sh*^ *n* = 6; P18-24 *Glra1*^+/+^ *n* = 7, *Glra1*^*sh*/*sh*^ *n* = 9), number of channels open at the peak current (P9-13 *Glra1*^+/+^ *n* = 4, *Glra1*^*sh*/*sh*^ *n* = 6; P18-24 *Glra1*^+/+^ *n* = 7, *Glra1*^*sh*/*sh*^ *n* = 9), and noise time constants (derived from power spectral density analysis) (P9-13 *Glra1*^+/+^ *n* = 10, *Glra1*^*sh*/*sh*^ *n* = 12; P18-24 *Glra1*^+/+^ *n* = 16, *Glra1*^*sh*/*sh*^ *n* = 15). Note that P_open_ was significantly lower in *Glra1*^*sh*/*sh*^ mice in the older age group compared to *Glra1*^+/+^ control. ^*^p < 0.05.

In order to investigate the functionality of glycinergic signaling at intact *in situ* synapses, we prepared brain stem slices from wild-type and *shaky* mice and performed whole-cell recordings from hypoglossal motoneurons (HM), which are rich in GlyR α1 subunit expression and important for respiration. Specifically, we asked how the shift in GlyR expression during postnatal development would affect the electrophysiological properties of synaptic and extrasynaptic GlyRs in HM neurons before (P9-13) and after onset (P18-24) of symptoms. In voltage-clamped HM neurons held at −70 mV, bath-applied glycine (10–1,000 μM) induced an inward shift of holding current when recorded with CsCl-filled pipettes (Figure 7A). In *Glra1*^+/+^ HMs, the dose-response relationships of glycine currents did not differ between the two age groups (*Glra1*^+/+^, P18-24, *n* = 11 from 6 mice; P9-13, *Glra1*^+/+^, *n* = 7 from 4 mice), with EC_50_ values of 270 μM in P9-13 HMs and 277 μM in P18-24 HMs, respectively (Figures 7B,C). In *Glra1*^*sh*/*sh*^ mice, however, EC_50_ values increased from 313 μM in P9-13 HMs to 384 μM in P18-24 HMs as *shaky* mice became symptomatic (Figures 7B,C; *Glra1*^*sh*/*sh*^, P18-24, *n* = 9 from 6 mice; P9-13, *n* = 8 from 5 mice). In addition to the reduced efficacy of externally applied glycine, we found that tonic inhibition by ambient glycine acting on extrasynaptic GlyRs was abrogated in slices from *Glra1*^*sh*/*sh*^ HMs (Figure 7D). By contrast, although the GlyR antagonist strychnine (2 μM) revealed substantial tonic inhibition in *Glra1*^+/+^ HMs through its effects on holding current and current variance, we failed to detect any change in these parameters upon strychnine application in HMs from P18-24 *Glra1*^*sh*/*sh*^ mice (Figures 7D,E; *Glra1*^+/+^, 24.2 ± 9.6 pA^2^, *n* = 6 from 4 mice; *Glra1*^*sh*/*sh*^, 1.8 ± 1.2 pA^2^, *n* = 6 from 5 mice; *p* = 0.043). We also performed stationary noise analysis to examine the amplitude and kinetics of the unitary events underlying the macroscopic glycine-evoked whole-cell currents at 100–300 μM glycine (Figures 8A–C). We did not detect any differences in unitary current amplitude, number of open channels, and noise time constants between *Glra1*^+/+^ and *Glra1*^*sh*/*sh*^ mice of either age group. By contrast, the open probability of channels was significantly reduced in HMs of older *shaky* mice compared to wild-type neurons of the same age.

**Figure 9:**
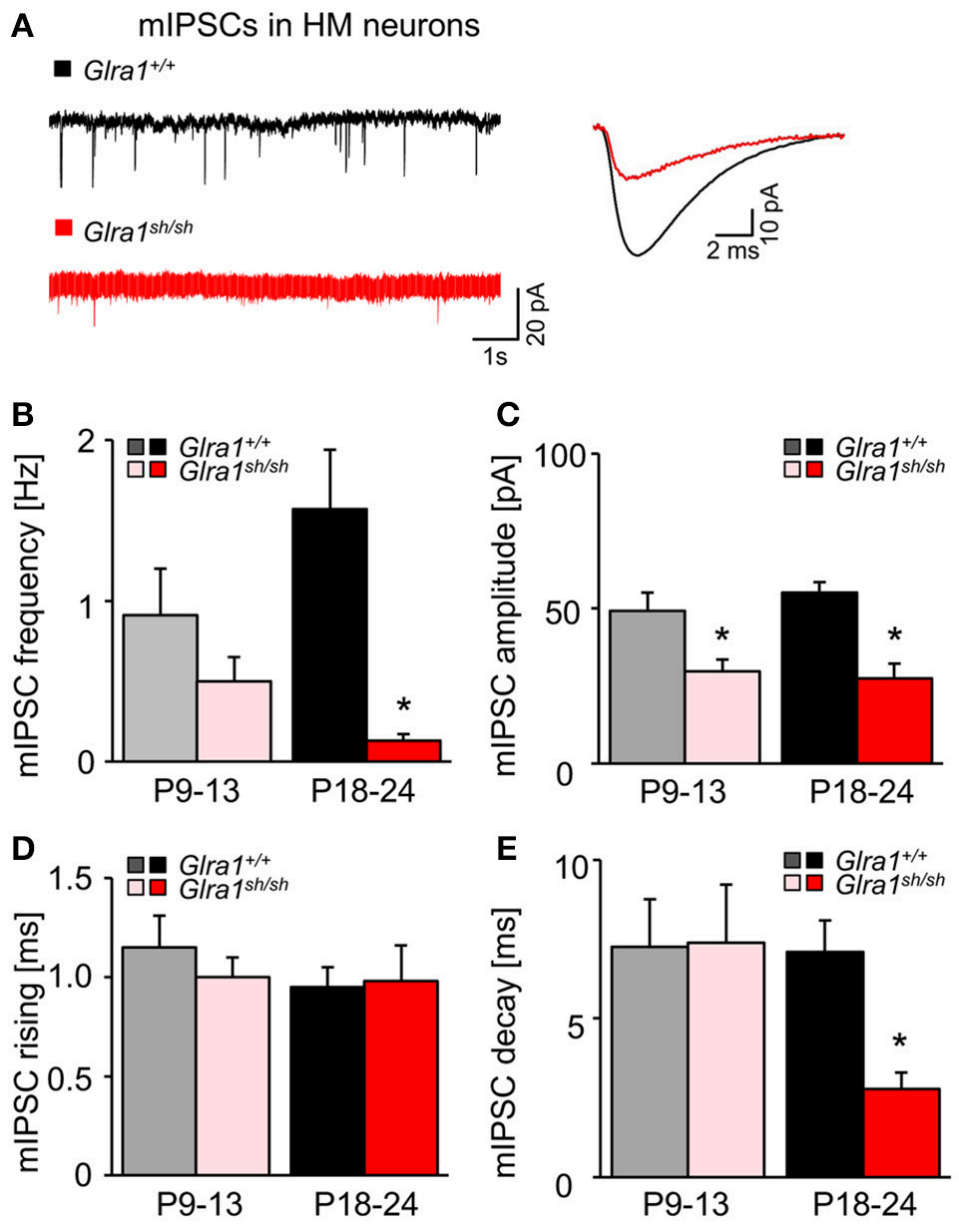
Developmental shift of glycinergic synaptic inhibition in *Glra1*^*sh*/*sh*^ HMs. **(A)** Representative traces from a *Glra1*^+/+^ HM and a *Glra1*^*sh*/*sh*^ HM at P18-24 illustrate miniature IPSCs (mIPSCs). Recordings were performed in the presence of TTX (1 μM), KA (2 mM) and bicuculline (10 μM). The superimposed traces on the right are the averaged mIPSCs from respective neurons on the left. **(B–E)** Bar diagrams summarize the changes of mIPSC kinetics in *Glra1*^*sh*/*sh*^ mice. Note that a change in mIPSC amplitude was already apparent in *Glra1*^*sh*/*sh*^ mice at P9-13 when the receptor replacement has already started but neuromotor symptoms have not yet appeared. Dramatic reductions in **(B)** mIPSC frequency (P9-13 *Glra1*^+/+^ *n* = 6, *Glra1*^*sh*/*sh*^ *n* = 6; P18-24 *Glra1*^+/+^ *n* = 9, *Glra1*^*sh*/*sh*^ *n* = 9), **(C)** amplitudes (P9-13 *Glra1*^+/+^ *n* = 6, *Glra1*^*sh*/*sh*^ *n* = 6; P18-24 *Glra1*^+/+^ *n* = 9, *Glra1*^*sh*/*sh*^ *n* = 9), and **(E)** decay (P9-13 *Glra1*^+/+^ *n* = 6, *Glra1*^*sh*/*sh*^ *n* = 6; P18-24 *Glra1*^+/+^ *n* = 10, *Glra1*^*sh*/*sh*^ *n* = 9) were uniformly observed in HMs from P18-24 *Glra1*^*sh*/*sh*^ mice. **(D)** Rise time constants (P9-13 *Glra1*^+/+^ *n* = 6, *Glra1*^*sh*/*sh*^ *n* = 6; P18-24 *Glra1*^+/+^ *n* = 10, *Glra1*^*sh*/*sh*^ *n* = 9). ^*^p < 0.05.

While current responses to bath-applied glycine or strychnine reflected predominantly the activation or suppression, respectively, of extrasynaptic GlyRs, we next examined the functionality of synaptic GlyRs mediating phasic inhibition in *Glra1*^+/+^ and *Glra1*^*sh*/*sh*^ HMs at both stages of postnatal development. In the presence of TTX (1 μM) and blockers of ionotropic glutamate receptors (KA, 2 mM) and GABA_A_ receptors (BIC, 10 μM), we monitored miniature inhibitory postsynaptic currents (mIPSCs) arising from spontaneous, action potential-independent release of glycine from presynaptic terminals. Compared to *Glra1*^+/+^ HMs, mIPSCs recorded in HMs from P18-24 *Glra1*^*sh*/*sh*^ mice showed a significant decrease in frequency, amplitude and decay time, but no change in rise time (Figure 9; *Glra1*^+/+^, *n* = 10 from 5 mice; *Glra1*^*sh*/*sh*^, *n* = 9 from 6 mice). Such strongly diminished mIPSCs have been also observed in PreBötzinger complex neurons of *shaky* mice (Schaefer et al., 2017), demonstrating a widespread deficiency of glycinergic inhibition in this mouse mutant. Interestingly, a decline in mIPSC amplitude was already evident in younger P9-13 mice (*Glra1*^*sh*/*sh*^ mice (*Glra1*^+/+^, *n* = 6 from 3 mice, *Glra1*^*sh*/*sh*^, *n* = 6 from 4 mice), arguing for a lack of compensation by other GlyR subunits at an early stage of the subunit switch.

## Discussion

The *shaky* mouse model, harboring the GlyR α1 subunit mutation Q177K, is the first *in vivo* model revealing that the integrity of the β8-β9 loop in the ECD is a key regulator of glycinergic signaling. The neuromotor phenotype of *shaky* mice is severe and incompatible with life. Functional impairment of GlyRs containing the α1 subunit in *shaky* mice is in line with structural data of the GlyRs illustrating conformational rearrangements with coupling of movements within the ECD (loop C, β1-2, β6-7) upon ligand-binding with elements of the ECD TMD interface (β10-pre-M1, the M2-3 loop) during GlyR channel gating processes (Du et al., 2015; Huang et al., 2017). Moreover, the GlyR α1 subunit structure suggested that the β8-β9 loop of the complementary subunit is localized underneath the ligand binding domain of two adjacent subunits, mediating rearrangements required to translate ligand binding into ion channel opening (Du et al., 2015).

In this study, we have focused on behavioral and functional disturbances in homozygous *shaky* mice *Glra1*^*sh*/*sh*^ during different stages of development: (i) after GlyR α1 subunit expression starts (P9-13) and (ii) after symptomatic disease onset (P18-24).

*Shaky* arose as a spontaneous mutation in a hybrid mouse background of C57BL6 and 129SvJ lines. Mutant mice become obvious due to their increased startle, muscle spasms and rigidity, tremors, abnormal gait starting at the age of postnatal day 14. These symptoms reflect the phenotype observed in several other startle disease mouse models, e.g., *cincinnati, nmf11, spasmodic, spastic* and *oscillator* (Schaefer et al., 2012). DNA sequence analysis of affected homozygous *shaky* mice (*Glra1*^*sh*/*sh*^), and wild-type control animals (*Glra1*^+/+^) from both mouse background lines identified a synonymous variant in exon 3 (c.T198C, N38N) and a missense mutation in exon 6 (c.C613A, p.Q177K) (Schaefer et al., 2017). A non-synonymous variant, e.g., in exon 3 (c.T198C, p.N38N) although present in both mouse backgrounds, does not necessarily mean that the base pair substitution at the DNA level does not contribute to the observed phenotype. For example, a single nucleotide polymorphism present in exon 6 of *spastic* mice that differed between the C57BL6 and B6C3Fe mouse lines used as background was identified as an important exonic splicing enhancer (ESE) site contributing to aberrant splicing underlying the *spastic* phenotype as well as the known insertion of a LINE1 element in intron 6 of the *Glrb* mouse model for startle disease (Becker et al., 2012). ESE analysis of both single nucleotide polymorphisms identified in *shaky* mice using the software ESEfinder 3.0 did not suggest the creation or destruction of an ESE in exon 3. However, the exon 6 was found to harbor an ESE in the wild-type sequence that is no longer present in the *shaky* sequence. However, no aberrant splicing of GlyR α1 subunit transcripts was detectable, excluding a splicing defect as a determinant in the pathomechanism of startle disease in the *shaky* mouse model.

Uncoordinated motor behavior and typical hind-limb clasping were the first visible phenotypic symptoms present at postnatal day 14. In addition, a longer time window for righting has been shown for *shaky* mice (Schaefer et al., 2017). All together, these symptoms are similar to symptoms reported in *cincinnati, oscillator, nmf11, spasmodic* and *spastic* mice (Buckwalter et al., 1994; Holland et al., 2006; Traka et al., 2006; Becker et al., 2012; Schaefer et al., 2017). Patients are symptomatically treated by diazepam, a positive allosteric modulator of GABA_A_ receptors (Praveen et al., 2001). Likewise, the activity pattern of affected homozygous *shaky* mice at P28 improved upon treatment with diazepam. Despite its utility in human startle disease, the molecular mechanism underlying the effectiveness of diazepam treatment is not completely understood.

In zebrafish, mutations within the glycinergic system lead to a loss of reciprocal inhibition between the left and right sides of the spinal cord, ending in the activation of motoneurons simultaneously on both sides (Grillner, 2003; Fetcho et al., 2008), following bilateral muscle activation and thus dorsal flexure of the body (Hirata et al., 2005). Mutant alleles *beo*^*tw38f*^ and *beo*^*mi106a*^ harbor different missense mutations in the zebrafish GlyR β subunit (L255R and R275H, respectively) that result in impaired escape responses. The R275H mutation in zebrafish GlyR β affects a highly conserved arginine residue prior to TM2. The corresponding mutation in the human GlyR α1 subunit, R252H, is known to accelerate degradation of GlyR α1 resulting in lack of GlyR ion channel function (Rea et al., 2002; Villmann et al., 2009). The analysis of the β8-β9 loop mutation GlyR α1^Q177K^ in zebrafish larvae revealed an abnormal escape response for some animals. The motor phenotype of the *Dhx37* zebrafish model was not recovered with GlyR α1^Q177K^ mRNA. The observed effect was mild compared to the murine phenotype and might be explained by the use of the MO-dhx37 model encoding a RNA helicase involved in GlyR α1, α3 and α4a subunit mRNA biogenesis and splicing. In zebrafish, five GlyR α subunits and two β subunit genes have been reported (David-Watine et al., 1999; Imboden et al., 2001; Hirata et al., 2005), arguing that GlyR α2, α4b and βa subunits that are not affected by MO-dhx37 treatment might contribute to less severity in the zebrafish model. These data, however further support the importance of the GlyR α1 β8-β9 loop for motor behavior shown in another animal model.

As a consequence of the neurological phenotype, significant physical changes e.g., body weight become apparent with a delay of 1–2 weeks following symptomatic onset in *shaky* mice. Differences in the initiation of disease symptoms between humans and mice have been attributed to differences in developmental regulation of GlyR expression of affected subunits. In rodents, GlyR α1 transcript has been first observed at E14 but with very weak signals. Clear signals are detected at P5 and continuously increase until P14 onwards (Malosio et al., 1991). At the protein level, it has been suggested that the developmental shift between neonatal and adult GlyR isoforms is completed after P21 (Becker et al., 1988). In neurons of the brain stem, e.g., PreBötC and HM GlyR α1 immunoreactivity was low between P2 and P11, with a slight peak at P7 and an additional peak that persisted from P12 until P21 (Liu and Wong-Riley, 2013). In humans, patients with *GLRA1* mutations suffer from neonatal hypertonia, an exaggerated startle reflex in response to tactile or acoustic stimuli and in some instances in life-threatening infantile apnea episodes immediately after birth arguing for a developmental switch of GlyR α2 to α1 subunits around birth (Davies et al., 2010).

Previously, we found the GlyR α1 protein in spinal cord and brain stem of wild-type, heterozygous and homozygous *shaky* mice at P7 (Schaefer et al., 2017). Here, the GlyR α1 subunit was clearly detectable at P7 in backcross experiments into the *spasmodic* mouse line. Hence, although the developmental shift between GlyR α1 and α2 subunits starts around P5 in rodents, *shaky* mice become phenotypically apparent at postnatal day 14 arguing at least for some compensation by other GlyRs or GABA_A_Rs within the second week of life. Such compensation by other GlyR α subunits cannot be attributed to an increase in the expression level of α2 or α3 subunits (Schaefer et al., 2017). Graham et al. demonstrated GABAergic compensation in *spastic* mice (which have lowered expression of the GlyR β subunit). In contrast, in *oscillator* mice no GABAergic compensation was observed (Graham et al., 2003). Recordings from PreBötC neurons of the brain stem at P18-24 did not reveal any differences in GABAergic mIPSCs between *Glra1*^*sh*/*sh*^ and *Glra1*^+/+^ animals. Thus, we excluded GABAergic compensation in *shaky* mice at least at this developmental stage (Schaefer et al., 2017).

Due to the enhanced muscle tone following acoustic and tactile trigger starting at P14, physical impairment, e.g., a reduction in body weight, becomes evident with an additional week delay. Thus, the questions arises what happens at the functional level of the GlyR following expression start of GlyR α1 subunit and after symptomatic disease onset? In *spastic* mice, a crosstalk between presynaptic and postsynaptic elements at different stages of development (P5 and P15) has been suggested to underlie the pathology (Muller et al., 2008).

Previously published data in PreBötC neurons of homozygous *shaky* mice showed reduced GlyR current amplitudes in P18-P24 old mice as well as faster ion channel closure when compared to wild-type neurons (Schaefer et al., 2017). Smaller current amplitudes of glycinergic mIPSCs have also been detected in *spastic, spasmodic*, and *oscillator* mice (Graham et al., 2003, 2006). Here, we focused on the functional analysis in hypoglossal motoneurons (HM) of the brain stem at two stages of development of *shaky* mice (P9-P13 and P18-P24). HM neurons are rich in glycinergic synapses and harbor similar motoneuron numbers in homozygous *shaky* mice compared to wild-type controls.

Patch-clamp recordings revealed significantly reduced amplitudes of glycinergic mIPSCs in the second postnatal week, i.e., about 1 week before apparent disease onset. This pre-symptomatic decline in current amplitude is likely to arise from the switch to the expression of mutated GlyR α1 which begins around P7 in *shaky* animals. In behavioral terms, the early deficits in GlyR function remain silent for about a week. However, once the full spectrum of GlyR deficits is achieved after the second postnatal week, compensatory mechanisms fail and symptoms appear. Functional compensation by other subunits seemed to assist during the second week of life, but failed at later developmental stages (3–6 weeks) when GlyR α2 is likely to have been completely replaced by GlyR α1 (Becker et al., 1988). This is in contrast to *spastic* mice harboring a *Glrb* mutation, which display a compensation by homomeric α1 subunit GlyRs at extrasynaptic loci (Graham et al., 2011). Corroborating and extending previous findings from PreBötC neurons of *shaky* mice (Schaefer et al., 2017), GlyRs of HMs from mutant mice with overt symptoms recorded between P18-24 showed the following electrophysiological aberrations when compared to their wild-type counterparts: (i) In addition to the reduction of mIPSC amplitude already present during the second postnatal week, we now also observed a dramatically reduced frequency and a much faster decay of mIPSCs. Such changes have also been observed in homozygous *spasmodic* mice during disease progression (Graham et al., 2006). (ii) Noise analysis showed that, while the number of GlyR channels and their unitary current amplitude remained unaffected, their open probability was significantly reduced. (iii) The dose-response relationship of GlyR currents to increasing concentrations of glycine exhibited a pronounced rightward shift leading to a significantly enhanced EC_50_ value, and finally, (iv) tonic glycinergic inhibition through extrasynaptic GlyRs was virtually abrogated. Thus, while the severe phenotype in *shaky* mice clearly results from non-functional postsynaptic α1β subunit GlyRs, defective extrasynaptic homomeric α1 subunit GlyRs may also contribute to the phenotype.

The decline in mIPSC frequency in brain stem slices of symptomatic *shaky* mice points to an additional impairment in glycinergic neurotransmission at the presynaptic site. Other studies have emphasized the contribution of presynaptic homomeric GlyR α1 complexes to startle disease (Xiong et al., 2014). In view of the high intracellular Cl^−^ concentration in presynaptic terminals, currents through presynaptic GlyRs are depolarizing. This might in turn activate voltage-dependent Ca^2+^ channels and elevate intracellular Ca^2+^, thereby promoting transmitter release, as shown for the calyx of Held and the spinal cord (Turecek and Trussell, 2001; Jeong et al., 2003). It seems therefore conceivable that, with functionally impaired homomeric GlyR α1^Q177K^ at the presynaptic site, transmitter release in *shaky* mice might be substantially affected.

Recent work also revealed novel roles of presynaptic GlyRs outside the brainstem. Functional presynaptic GlyRs were demonstrated at mossy fiber terminals in the hippocampus at mossy fiber terminals mainly during postnatal development (Kubota et al., 2010). Using gain-of-function GlyR α3 subunit mutants, their impact on hippocampal network activity and related behaviors and diseases was established (Winkelmann et al., 2014; Çaliskan et al., 2016). Since glycinergic effects in the hippocampus are likely to be mediated by GlyRs containing α2 and α3, but not α1 subunits, the *shaky* α1 mutant is unlikely to alter the presynaptic actions of glycine in the hippocampus.

Presynaptic compensation by other GlyR subtypes might maintain glycinergic inhibition to some extent during the second postnatal week until homomeric neonatal GlyRs are down-regulated to make way for adult α1β GlyRs that are no longer able to sustain glycinergic inhibition.

In summary, we have demonstrated that the *shaky* mutation in the GlyR α1 subunit results in defective synaptic integration, incomplete compensation and functional disturbances that are incompatible with survival. We have also provided novel insights into the pathogenesis of startle disease by demonstrating that motor disturbances and functional deficits at synapses can be observed prior to the onset of severe symptoms. Lastly, we have shown the deficits in presynaptic GlyR function are also apparent in *shaky* mice, suggesting that deficits in presynaptic glycine release, as well as postsynaptic GlyR function, contribute to startle disease.

## Author contributions

AB performed transcript analysis. AB and NS conducted animal behavioral tests. HH conducted zebrafish studies. SL and RH performed sequencing analysis of genomic DNA. NS conducted mouse backcrosses with other GlyR mutant mouse lines. NS conducted counting of motoneurons. FZ and JvB conducted patch clamp recordings in brain stem slices. NS and CV performed protein analyses from mouse tissues. NS, CV, AB, FZ, JvB, and HH performed data analyses. NS, CP, CA, RH, and CV participated in manuscript writing. CV initiated, designed and supervised the project.

### Conflict of interest statement

The authors declare that the research was conducted in the absence of any commercial or financial relationships that could be construed as a potential conflict of interest.

## Abbreviations

GlyRs: Glycine receptors
GlyT2: glycine transporter 2
CLRs: Cys-loop receptors
ECDs: extracellular domains
*Glra1* ^+/+^: wild type
*Glra1* ^*sh*/*sh*^: *shaky* mice
mIPSCs: miniature inhibitory postsynaptic currents
aCSF: artificial cerebrospinal fluid
KA: kynurenic acid
BIC: bicuculline
TTX: tetrodotoxin
SNA: Stationary noise analysis
HM: hypoglossal motoneurons
